# Management of breast cancer in an Asian man with post-traumatic stress disorder: a case report

**DOI:** 10.1186/s13256-016-0864-0

**Published:** 2016-03-29

**Authors:** Faaizah Patel, Rajgopal Achuthan, Lucie Hyklova, Andrew M. Hanby, Valerie Speirs

**Affiliations:** 1 Breast Unit, St James’s University Hospital, Leeds, UK; 2 Leeds Institute of Cancer and Pathology, University of Leeds, Leeds, LS9 7TF UK

**Keywords:** Male breast cancer, Ethnicity, Afghan, PTSD

## Abstract

**Background:**

Migration to the UK has increased considerably, which is reflected in the diverse multicultural population which includes asylum seekers and economic migrants. Differences in ethnic and cultural values between the host and newcomer populations could impact on effective health care provision, especially in gender-biased conditions such as breast cancer. Breast cancer is rare in men and the diagnosis is often met with disbelief. This case report describes an unusual case of breast cancer in an Afghan man who is an asylum seeker of Asian ethnic origin.

**Case presentation:**

A focused ethnographic case study and in-depth interview was used to gain qualitative data and insight into the personal experiences of a male Afghan asylum seeker, age unknown (estimated to be in his 30s), with post-traumatic stress disorder who was electively admitted into hospital for the investigation of a suspicious lump in his left breast, which was subsequently found to be breast cancer. He was extremely reluctant to accept a breast cancer diagnosis and initially would not consent to any treatment, preferring to seek further opinion. During consultation with various members of the breast team he continually declined to accept the diagnosis and felt there was an error in the investigative protocol. Through the involvement of a Muslim nurse, fluent in Urdu and knowledgeable of the Afghan culture and religious background, we learned about his experiences and feelings; he opened up to her about his experiences in Afghanistan, detailing his experiences of trauma as a result of war, and disclosing that he had been diagnosed as having post-traumatic stress disorder by his physician. He saw breast cancer as a “woman’s disease” which deeply affected his feelings of masculinity and left him feeling vulnerable.

**Conclusions:**

While sensitivity is undoubtedly required when diagnosing gender-biased conditions such as breast cancer in men, our experience showed this is exacerbated in ethnic minority groups where language barriers often exist and awareness of cultural differences is required. Awareness of the possibility of post-traumatic stress disorder in migrant populations from conflict-torn areas is also recommended during consultation.

## Background

Through various national campaigns there is now a high level of general awareness of breast cancer in women. Male breast cancer however, is still comparatively unknown by the general public, and some health care and social care professionals. The incidence of breast cancer is rising in men [1], but still remains rare, with approximately 350 cases diagnosed in the UK annually, compared to some 50,000 women [2].

Detection, treatment and management strategies for breast cancer are gender biased in that they are currently based exclusively on those used for women. This includes national campaigns to raise awareness of breast cancer, such as the pink ribbon which further reinforces gender stereotyping of the disease. Topics discussed in support groups, the literature provided for the educational needs of those with breast cancer, and websites that are used as a means of informing patients, all include prosthesis, bra fitting services and advice, as well as breast reconstruction information, which are all directed toward women [3]. The psychological impact of gender bias cannot be underestimated [4, 5].

The deficiency of patient-specific care for men with breast cancer [6] is exacerbated in ethnic minorities where language and cultural issues may present additional barriers. Immigration into the UK has increased significantly over the past decade [7], resulting in a diverse multicultural population which also includes asylum seekers. Data from the European Asylum Support Office showed that in 2011, 28,000 Afghans applied for asylum in the European Union, the highest number in the decade, a consequence of the political situation and conflict in Afghanistan [8]. Studies from mental health research indicate that asylum seekers, particularly those from war-torn countries, are likely to develop post-traumatic stress disorder (PTSD) as a result of traumatic events such as military combat, violent personal assault (for example rape), natural disasters, or other threats to life [9, 10].

We document our experience of the management of an Afghan man who presented to our Breast Unit with a suspicious lump in his left breast, which was subsequently diagnosed as breast cancer.

## Case presentation

An Asian man estimated to be in his 30s presented to our Breast Clinic via his physician with a suspicious lump in his left breast. An ultrasound scan demonstrated a 30×13 mm irregular mass in his left breast above the nipple areolar complex. The mass looked malignant and abnormal lymph nodes were seen in his left axilla. A histopathological diagnosis of a core biopsy confirmed a grade 1 invasive mucinous carcinoma that was estrogen receptor (ER)-positive, progesterone receptor-positive, and human epidermal growth factor receptor 2-negative. A core biopsy of the suspicious axillary lymph node confirmed this was benign. The results were discussed in the Breast Multi-disciplinary Team meeting which recommended that the patient should have a mastectomy and sentinel lymph node biopsy.

The results of the core biopsies and details of surgery were explained to the patient as well as further adjuvant treatments by a male breast oncoplastic surgeon and female breast care nurse. The patient was extremely reluctant to accept the diagnosis of breast cancer. He requested copies of all the results including his imaging and histopathology report. He understood the information that he was given but struggled to come to terms with it and declared he did not believe the diagnosis of breast cancer. He stated he would seek a second and possibly third opinion and that he would not consent to any treatment at this stage. He also informed the consultant that he would refuse any further appointments to the Breast Clinic but may contact his physician at a later date to discuss his diagnosis. The consultant contacted the patient’s physician by telephone and informed the physician about the consultation. The physician kindly agreed to see the patient and discuss things further. A further out-patient appointment was also offered to the patient.

The patient re-attended the Breast Clinic 19 days later and was seen by a female registrar and a female breast care nurse. Subsequently, he had numerous discussions with the breast care nurses. He continually declined the diagnosis of breast cancer and felt there was an error in the investigative protocol. The registrar offered him repeat scans/biopsies to see if this would help him and offered him a referral to see another breast surgeon in the same hospital or one nearby; although, he accepted the offers he stated he may only pursue this after his upcoming examinations, as he was a student. The registrar’s main concern was the patient’s reluctance to proceed with any form of treatment for the diagnosed breast cancer. As the tumor was ER positive, the registrar offered the patient the option of endocrine therapy; however, he declined this too.

The registrar stated the patient communicated very well and clearly with regards to all the information that had been provided but the difficulty lay in his refusal to accept the diagnosis. The patient again requested a copy of all his results, which he was given; he stated he would contact his physician soon to discuss seeking a second opinion from another breast surgeon. He was given another appointment to be held 3 weeks later as well as all necessary contact details to stay in touch. He was reassured that should he change his mind at any point, the breast team was there for him.

He contacted one of the breast care nurses and decided to have a further discussion with the breast team and was seen in clinic again. When seen by his original consultant, he stated he had come to terms with his diagnosis and decided to proceed with surgery. He was very anxious with regards to survival and he was told by the consultant that with the treatments available the chances of curing the cancer would be high. He was comfortable with the fact that he would need a mastectomy and sentinel lymph node biopsy, and he accepted the fact that he would possibly require further surgery if the sentinel lymph node biopsy was positive. He wanted to have his surgery done as soon as there was availability to ease his anxiety. A pre-assessment was arranged and his surgery was scheduled for the following week.

On the morning of his surgery he was approached by a Muslim nurse from the breast team. She was identifying patients who would be agreeable to their surplus pathology tissue being stored for research once the clinical need was met. Being fluent in Urdu and knowledgeable of the Afghan culture and religious background she quickly built a good rapport with the patient. He soon opened up to her about his experiences both in Afghanistan and England. He detailed his experiences of trauma as a result of the war in Afghanistan: “I witnessed people’s limbs blown apart because of bombings; their families had no hospital to take them to because there’s no infrastructure like here. Death, so much death, everywhere I went there was death…. They buried the dead in mass graves, all these bodies thrown into a big hole in the ground…. Now I’m terrified of death.” He had no family in England as they lived in Afghanistan, he stated: “I call them now and then…no, I don’t want to tell them about my breast cancer.” He was a student with few friends and lived a life of isolation. He had felt very vulnerable since his diagnosis and saw breast cancer as a “woman’s disease” which deeply affected his feelings of masculinity. As the interview progressed, he disclosed that he had been diagnosed as having PTSD by his physician. He was initially on medication for this but it had been decided to stop treatment because of the severity of the side effects. Neither his pre-assessment document nor the hospital doctors’ notes included his history of PTSD presumably as a result of a breakdown in communication from the physician to the hospital and the patient not informing staff when asked.

It also transpired that his birthplace and upbringing were in a rural isolated area of Afghanistan without health care services, where the population was predominantly illiterate. He did not know his birthdate or age as it was not common practice for these details to be documented. He stated he was unaware of any family history of genetic conditions or causes of death (apart from bombings).

He informed the nurse that he had associated cancer with death. When the nurse enquired if this association may have been linked to his PTSD and fear/phobia of death, he replied: “hmm…probably, I don’t want to die, I’ve seen too much death.” The nurse empathized with him as he told her of his journey filled with trauma and suffering. Her counselling and pastoral support from an Islamic perspective allowed him to view his illnesses, breast cancer, and PTSD differently. He found great comfort and relief in “finding a person I can trust” and he gradually reduced his anxiety levels. By the end of their discussion he realized that surgery was the best option for him and, when asked by the nurse, he consented to contribute tissue and blood to a Breast Tissue Bank to be used in research.

The nurse maintained this moral and psychosocial support in addition to providing continuity of care by visiting him again following his surgery. She managed his discharge process and provided details of drain care, risk of infection, wound management, arm exercises and physiotherapy, analgesia, body image, and scar healing. Contacts in the hospital and community setting for psychosocial support were additionally provided.

He was commenced on systemic endocrine therapy with tamoxifen and remains well.

## Conclusions

Previous studies have demonstrated that a diagnosis of breast cancer in men is often met with disbelief and there is often limited awareness of male breast cancer which still remains relatively unknown to the general public [11]. This case report has shown the difficulty that this can cause in developing an optimal treatment pathway, which can be further compounded in an ethnic minority group where language and culture add additional complexity. In this case a further layer was added by virtue of exposure to war with attendant PTSD. As a consequence of the influx of immigrants from diverse ethnic backgrounds who are accessing and utilizing the UK National Health Service, it is inevitable that there will be more patients where language, culture and often conflict create complex treatment scenarios requiring specialist transcultural skills to fully meet patient needs. As demonstrated here, the emotional support which was provided by a nurse fluent in the patient’s native language and who was familiar with his ethnic and cultural background helped the patient to come to terms with this difficult diagnosis. Awareness of cultural differences is required when treating ethnic minority groups and asylum seekers with gender-biased conditions such as breast cancer. In addition, health care operatives need to be alert to the possibility of PTSD in migrant populations from conflict-torn areas and consideration of awareness courses for staff in hospitals where such migrant populations are treated should be given.

## Consent for publication

Written informed consent was obtained from the patient for publication of this case report and any accompanying images. A copy of the written consent is available for review by the Editor-in-Chief of this journal.

## Ethics approval

Ethical approval was obtained by Leeds (East) Research Ethics Committee (09/H1306/108).

## Abbreviations

ER: estrogen receptor
PTSD: post-traumatic stress disorder
